# Literary Notes

**Published:** 1897-11

**Authors:** 


### Literary Notes.

The first annual report of the state board of medical registration and examination, of Ohio, is an interesting and instructive document of 179 octavo pages, besides thirty-six pages of preliminary matter. The latter record the proceedings of the board at its several meetings, while the former contain the statutes of Ohio relating to the practice of medicine and surgery now in force, as well as those that are obsolete; the official register of physicians by counties; the index to the official register and an alphabetical list of midwives. The report indicates an enormous amount of work performed by the board during its first year. It is presumed that it will be less onerous during succeeding years. The report should be in possession of every state examining board.

---

The *Bulletin* of the Harvard Alumni Association, Number 11, contains the report of the seventh annual meeting, held at Boston, June 29, 1897. Besides routine matters it contains the several speeches made at the annual dinner and much other interesting material. Typographically it is a model brochure and should serve to stimulate the publication of their proceedings by other alumni associations.

---

The *National Medical Review*, of Washington, D. C., has passed into the editorial control of Drs. Thomas E. McArdle and George W. Johnston, both capable and experienced physicians and medical writers. The former editor, Dr. Charles H. Stowell, has gone to Boston to engage in literary work. We bespeak for the *Review* a continuation of its success on enlarged and broadened lines.

---

The *Medical Council*, of Philadelphia, has changed its address to the northeast corner of 12th and Walnut streets.

The *National Hospital and Sanitarium Record*, published at Detroit, Mich., is one of the additions to the periodic literature made during the month of September.

---

*Edwards's Journal of Health*, edited and published at Atlantic City, N. J., by Dr. Joseph F. Edwards, issued its first number in October. The editor was formerly attached to the *Annals of Hygiene* in a similar capacity and promises that this new venture will even surpass the *Annals* in interest and value. The first number certainly presents a fine appearance.

